# Continuity of care in suicide prevention: current status and future directions

**DOI:** 10.3389/fpubh.2023.1266717

**Published:** 2024-01-08

**Authors:** Shay Arnon, Golan Shahar, Anat Brunstein Klomek

**Affiliations:** ^1^Baruch Ivcher School of Psychology, Reichman University, Herzliya, Israel; ^2^Department of Psychology, Ben-Gurion University of the Negev, Be'er Sheva, Israel

**Keywords:** continuity of care, chain of care, suicidality, suicide prevention, suicide

## Abstract

**Introduction:**

Continuity of Care (CoC) is central to suicide prevention. The present study aims to review contemporary definitions, operationalization in research, and key components of CoC in the prevention of suicide.

**Methods:**

The present study is a narrative review. A thorough search of available literature on CoC and suicidality was conducted. Studies published between 1995 and 2021 were reviewed and selected based on relevance to CoC and suicidality. Selected research was subsequently summarized to outline definitions of CoC, its operationalization in research, and key components for suicide prevention.

**Results:**

The definition, measurement, and operationalization of CoC in suicide prevention varies tremendously, derailing clinical practice. Key elements of CoC identified across the literature include (1) CoC across multiple levels of care, (2) the role of primary care providers and case managers in CoC of suicidal patients, (3) the importance of follow up contact with suicidal patients post-treatment, and (4) the role of national and institutional guidelines for CoC of suicidal patients. Limitations: There is a dearth of randomized controlled trials and insufficient evidence on specific populations.

**Conclusion:**

CoC refers to a wide, complex concept that must be broken down into specific categories that can provide more nuanced guidance of research and clinical implications.

## Introduction

Contemporary investigations have identified the crucial role of continuity of care (CoC) in suicide prevention and on the mental health outcomes of patients at risk of suicidality (1, 2), In April 2021, Itzhak Saidian set himself on fire, both as a suicide attempt and as a protest against the institutional neglect directed by the Israeli Ministry of Defense against war veterans suffering from post-traumatic stress disorder (PTSD). Saidian’s act increased particular attention to the concept of CoC in suicide prevention. According to the Israeli media (3), the absence of CoC played a key role in the tragic outcome of Saidian’s treatment. Indeed, the literature identifies disruptions of CoC as barriers to the prevention of suicide, which is a global health concern (1, 2, 4). On the contrary, promotion of continuity is considered fundamental for any national initiative looking to address the phenomenon of suicidality (1, 2).

Further empirical studies suggest poor or lacking CoC is related to elevated risk of hospital readmission and attempted and completed suicide, whereas better CoC appears to be protective against suicidality (5–10). A recent study estimated about 23% of suicides were partially attributed to CoC issues such as admission difficulties, patient refusal of services, limited services offered by a continuing care team, abrupt termination of services, lack of community follow-up, poor transition between services, and the loss of or change in case manager (11). Integrated models of care coordination are especially critical for high-risk patients, such as those at risk of suicide, in which the effective and immediate transition of treatment and care-related information, as well as prevention of treatment fall-out, can have a life-saving consequences (12, 13). CoC impacts the ability to continuously screen, assess and monitor for suicidality and fluctuations in suicidal risk, as well as provide and maintain the efficacy of suicide interventions and suicide prevention strategies over time (e.g., safety plan interventions and other suicide reduction strategies), thereby reducing suicide risk and suicide rates (14).

However, what exactly do practitioners, researchers and policy makers mean when they refer to CoC? To date, highly variable definitions exist for CoC, obscuring this crucial concept and leading to discrepancies in its operationalization in research and practice. The present article provides a narrative review of extant literature on the definitions, operationalization, and role of CoC in preventing suicide and offers a detailed discussion of necessary actions to better understand how to employ the concept of CoC in research and clinical care.

## Methods

Due to the interdisciplinary nature of our topic, we selected Google Scholar as our search engine as it is the most comprehensive one, inclusive of other engines such as PubMed, PsychInfo, etc. Studies published between 1995 and 2021, incorporating the following key words were included in this study: chain of care, continuity of care, suicidality, suicide, attempts, ideation, depression, self-harm, follow up, primary care, case management\manager, care management\manager, medication adherence, and treatment adherence. Studies conducted among children and adolescents were not included in this review because of their unique developmental nature and will be reviewed in another article.

Identified literature (43 articles) was subsequently summarized to identify types and definitions of CoC, current operationalization of CoC in research, and to outline key characteristics paramount for CoC in suicide prevention: (1) CoC across different levels of care (2) the role *of primary care provider* (PCP) and\or *case managers* (CM) in the care of suicidal patients, (3) *follow up* of suicidal patients, and (4) and *national and institutional guidelines* for CoC. The description of participants, design and CoC relevance for studies included in this review appears in Table 1.

**Table 1:** Summary of literature on CoC and suicidality.

Theme	Authors	Sample	Study design	CoC Relevance
**CoC across multiple levels of care**				
	American Psychiatric Association Task Force on Psychiatric Emergency Services (15)		Review	
	Choi et al. (5)	18,702 psychiatric inpatients	Quasi-experimental; nested case–control study	CoC was measured by the Continuity of Care Index [COCI; Bice and Boxerman (16)] from the time of hospital discharge until readmission or death.
	Desai et al. (7)	121,933 patients discharged from psychiatric inpatient units in the Veterans Affairs health care system	Quasi-experimental; prospective mortality study	Continuity of outpatient care post-discharge was assessed by the number of 2-month periods in the 6 months after discharge in which the patient had at least two outpatient visits related to the primary discharge diagnosis.
	Kim et al. (8)	48,558 patients diagnosed with unipolar\bipolar depression at risk of hospitalization.	Quasi-experimental;	Measured CoC for the first year of outpatient visits after receiving unipolar\bipolar depression diagnosis based on the usual provider of care index.
	King et al. (6)	234 psychiatric participants who died by suicide within 1 year of hospital discharge; 431 controls.	Quasi-experimental; retrospective case–control study	CoC was assessed by the number of days a patient had been “out of contact” (“the interval between date of a missed appointment\self-discharge and date of next point of contact”) and changed in key personnel (e.g., keyworker, out-patient doctor) after discharge.
	Knesper (12)		Review	Report commissioned by the Suicide Prevention Resource Center (SPRC) in collaboration with the Substance Abuse and Mental Health Services Administration (SAMHSA) regarding continuity of care for suicide prevention and research.
	Meehan et al. (17)	4,859 cases who had contact with mental health services during the 12 months prior to suicide	Quasi-experimental; national clinical survey	Contact with services at time of suicide, likelihood of continuing to community care, identification of characteristics associated with disruption in patterns of care.
	Mehlum et al. (18)	911 patients hospitalized for attempted suicide	Quasi-experimental; Prospective study.	The study examined the role of organizational changes (i.e., change in catchment area, increase in distance between hospital and local communities it served) on implementation of CoC models.
	Mehlum et al. (19)	48 hospitals	Quasi-experimental; qualitative study	Comparison of hospitals that implemented CoC programs for suicide attempters with other emergency departments.
	Mehlum and Mork (9)		Book chapter	Propose numerous requisites for ensuring CoC of suicidal patients, highlight findings from studies on the Norwegian CoC model
	Mork et al. (20)	47 informants from general hospitals, community health services	Quasi-experimental; qualitative study	Outcome measure of interview was whether community health services had CoC structures based on established criteria; impact of establishing CoC structure.
	Oordt et al. (21)		Review	
	Riblet et al. (22)	78 randomized controlled trials comparing suicide prevention strategies with control conditions	Meta-analysis	
	Riblet et al. (23)	16 patients hospitalized at a Veterans Affairs inpatient mental health unit., clinically fit to be discharged to outpatient care	Quasi-experimental; mixed methods	Measures of outpatient mental health treatment (i.e., total number of visits between discharge and 3-month follow up), regularity of outpatient care (number of consecutive months in first 3-month post-discharge in which the patient had at least one treatment visit), continuity of mental health treatment across intra-organizational boundaries (i.e., whether patient received outpatient mental health treatment within the first month post-discharge).
	Rossow et al. (24)	430 Norwegian municipalities	Quasi-experimental	Evaluating whether CoC reduces suicide rates and whether suicides rates decrease more in areas where CoC models have been implemented compared to other areas.
	Troister et al. (10)	A review of 28 articles that examined predictors of suicide within 1 year post-discharge	Review	
	Uijen et al. (25)	264 patients at risk for depression, 327 heart failure patients	Quasi-experimental; exploratory study	CoC was measured using a patient questionnaire assessing number of care providers contact and personal continuity, team continuity and collaboration, and continuity and collaboration with external care providers.
**Role of PCP and CM in the care of suicidal patients**				
	Bauer et al. (26)	11,015 adults enrolled in mental health integration program	Quasi-experimental; Observational analysis	Care manager contact within primary care-based mental health program.
	Bruce et al. (27)	598 elders diagnosed with depression	Randomized controlled trial	PROSPECT intervention emphasizing role of physician knowledge in treating depression in primary care setting and the employment of depression care managers to assist physicians in depression assessment, treatment recommendations, monitoring of clinical status and providing of follow up. Care managers interacted with patients in person or by phone at scheduled intervals of when clinically necessary in order to monitor depressive symptoms, adverse effects of medication and treatment adherence.
	Carrigan and Lynch (28)		Review	
	De Leo et al. (29)	261 suicide cases and 182 sudden death (control) cases	Quasi-experimental; case control study	Contact with general practitioners, psychiatrist and other health professionals in the 3 months prior to suicide (or death).
	Gensichen (30)	626 patients diagnosed with major depression.	Cluster randomized controlled trial	Case management intervention consisting of structure phone interviews to monitor depressive symptoms and support medication adherence, with feedback to family physician.
	Luoma et al. (31)	40 studies examining rates of patient contact with primary care and mental health care professionals prior to committing suicide.	Review	
	Schulberg et al. (32)		Review	
	Udo et al. (33)	20 depressed patients who experienced care manager contact	Quasi-experiemntal; qualitative explorative study	Patient experience having contact with care managers within primary care settings.
	Unützer et al. (34)	1801 older patients diagnosed with major depression, dysthymic disorder or both	Randomized controlled trial	IMPACT intervention included access to a depression care manager and a primary care expert who offered education, care management, support for medication management and brief psychotherapy.
**Patient follow up**				
	Brodsky et al. (14)		Review	Review of suicide prevention research and its role in implementation of evidence-based practices (e.g., Assess, Intervene and Monitor for Suicide Prevention; AIM-SP) to manage suicide risk in clinical setting.
	Cedereke et al. (35)	216 suicide attempters	Randomized controlled trial.	Phone intervention included two phone contacts emphasizing and encouraging treatment adherence.
	Fleischmann (36)	1,867 suicide attempters	Randomized controlled trial	Brief intervention and contact program (BIC) included a 1 h information session as soon as possible after discharge and nine follow up contacts, in addition to treatment provided as usual.
	Inagaki et al. (37)	Studies examining interventions to prevent repeat suicidal behavior among patients who presented to emergency departments due to a suicide attempt.	Review; Meta-analysis	
	Joyce et al. (38)	1,650 youths with a new episode of depression who initiated antidepressant treatment	Quasi-experiemntal; retrospective, longitudinal cohort design	CoC was evaluated as at least three follow-up appointments in the 12 weeks after first antidepressant prescription fill with at least one of those visits being with the prescribing physician. Follow up visits included encounters with mental health professionals.
	Katon et al. (39)	386 patients with recurrent major depression or dysthymia who recovered after 9 weeks of antidepressant treatment by PCP.	Randomized controlled trial	Intervention condition included 2 primary care visits with a depression specialist and 3 phone visits over 1 year, targeting treatment adherence, recognition and monitoring of symptoms and devising of a relapse prevention plan.
	Labouliere et al. (40)	73,732 Medicaid enrolled patients	Review	Assess, Intervene, Monitor for suicide prevention (AIM-SP) providing screening and risk assessment for high risk patients on a Suicide Safer Care Pathway (SSCP) with specialized care and increased contact\outreach (explicit procedures for maintaining CoC if patients miss appointments, etc).
	Lizardi and Stanley (41)	Studies investigating treatment engagement of suicide attempters.	Review	
	Luxton et al. (42)		Review	A review of the evidence for effectiveness of suicide prevention interventions that involve follow-up contacts with patients.
	Simon et al. (43)	208 patients starting antidepressant treatment for depression.	Randomized controlled trial	Intervention included three online care management contacts with a psychiatric nurse to assess severity of depression and facilitate follow up care as needed. Feedback was communicated online both to the patient and physician.
	Solberg et al. (44)		Review	Review of follow up systems in clinical practices for management of depressed patients in primary care.
	Stanley et al. (45)	100 participants presenting to the emergency department for suicide-related concern.	Quasi-experimental; qualitative interview study	The intervention included safety planning intervention (SPI) to provide coping skills and social support that could be employed in case of suicidal ideation and structured follow up (SFU) including contact within 1 week of emergency department visit and additional calls until patient attended his first outpatient appointment and a final call to assess satisfaction with outpatient care.
	Stanley et al. (46)	1,186 patients in intervention group, 454 controls.	Quasi-experimental; Cohort comparison study	Safety planning intervention which provides patients with coping skills and strategies to deal with suicidal ideation and behavior along with phone follow up (at least 2 calls) to review and revise the safety plan and encourage treatment engagement.
	Zalsman et al. (2)		Systematic Review	Review of studies assessing evidence of suicide prevention interventions.
**National and institutional guidelines for CoC**				
	American Psychiatric Association (47)		Review	A review of practice guidelines outlined by the American Psychiatric Association for the assessment and treatment of patients with suicidal behaviors.
	Department of Veterans Affairs Office of Inspector General (48)		Review	Review assessing the implementation of action items relevant for suicide prevention within the Veterans Health Administration (VHA’s) Mental Health Strategic Plan (MHSP), summarize findings and recommendations related to addressing of suicide prevention.

## Results

### Present definitions of CoC across the literature

Broadly, CoC refers to an integrated model of overall care coordination between different patient services and activities (e.g., hospitals and community services) which facilitates the delivery of appropriate aid to the patient (12, 13). Coordination of care includes the smooth transition between a patient’s providers and effective transmission of relevant information for the patient’s treatment and management (12, 13). However, a thorough investigation of the literature highlights present studies refer to a diversity of types of CoC (presented in Table 2). For example, some authors [e.g., (5, 49) divide CoC into informational continuity, relational\relationship continuity, and management continuity]. Others, employ the terms provider continuity and contact continuity (6). Lastly, Uijen et al. (25) refer to personal continuity, team continuity, and cross-boundary continuity. Evidently, the mixture of sub-definitions of CoC make it challenging to compare and translate these into integrated clinical guidelines.

**Table 2:** Continuity of care sub-definitions.

Subtype	Definition
Informational Continuity (5, 49)	Continuity in transmission of information between providers.
Provider continuity (6)	Continuity of the professional caregiver and whether there had been changes in key personnel due to professional provider being on leave or leaving.
Relational\Relationship Continuity (5, 49)	Continuity and stability of the relationship between the patient and the provider.
Personal Continuity (25)	Number of care providers a patient contacted (i.e., inside and outside of general practice) for their condition.
Contact Continuity (6)	Continuity of contact, i.e., whether there had been a break in care contact (absence from treatment appointments, disruption or lack of communication\response).
Management continuity (5, 49)	Continuity in the care of patients (i.e., consistency and flexibility).
Team continuity (25)	Intra-organizational collaboration between care providers within the treating practice or institution.
Cross-boundary continuity (25)	Inter-organizational collaboration between general practitioners and outside care providers (i.e., additional mental health or community services).

### Operationalization of CoC across the literature

In line with the variability of subtypes of CoC, studies documenting the relationship between CoC and mental health outcomes of patients at-risk for suicide also differ in their operationalization of the concept. Some studies measured CoC using the Continuity of Care Index [COCI (16)] which calculates an individual’s total number of visits with a single group of referred providers within a specific time-period (5). This is determined by quantifying the total number of visits, the number of visits to the specific provider and the number of unreferred providers (5, 16). Another study among psychiatric inpatients from Department of Veterans Affairs (VA) hospitals defined CoC as the number of 2-month periods in the half year post-discharge in which the patient had at least two outpatient visits to address his primary discharge diagnosis (7). In a study among patients diagnosed with unipolar and bipolar depression (8), CoC was measured using the Usual Provider of Care Index [UPC (50)] which quantifies the percentage of a patient’s number of outpatient visits to the most frequently seen provider out of his total number of visits. Lastly, in a retrospective case–control study looking to identify factors associated with increased suicide risk among patients who died within 1 year of hospital discharge, King et al. (6) measured CoC as the timeframe between a missed appointment or self-discharge and the date of the following contact (i.e., how long a patient had been “out of contact”). These studies join others in evidencing tremendous variability in the measurement of CoC, stemming from the absence of a unified conceptualization and definition of CoC in suicide prevention.

### Key components of CoC

#### CoC across levels of care

A prominent body of research concerns CoC across multiple levels of care, such as following patients’ discharge from inpatient care (5–7, 12, 23) and following ED\ER visits without hospitalization (12, 15, 18–20). These studies and their findings are summarized in Table 1. Recommended guidelines and conceptualization identify the following components for CoC following hospital\ED\ER admissions (5, 7, 9, 12, 15, 18–21, 23, 24):

1. *Appointed Authority:* designation of a suicide attempt service group or coordinator who would be responsible for implementation and evaluation of established guidelines.
2. *Monitoring*: of hospital admissions related to suicide attempts.
3. *Training*: staff to conduct systematic psychosocial and suicide risk assessments for suicide attempters.
4. *Written Protocol*: establishment of written procedures and responsibilities for suicide management and aftercare.
5. *Implementation:* of agreed management plans for suicidal patients.
6. *Structured collaboration:* between hospitals and aftercare providers to ensure adequate follow up treatment.

Smooth transition to outpatient care is recommended to be achieved by scheduling next-day appointments following discharge (or up to a maximum of one-week following discharge), by making reminder calls and by having active, timely follow up and effective communication between providers and care centers (6, 7, 12). If scheduling immediate care is impossible, present research recommends patients have some type of access to a psychiatrist until the scheduled outpatient appointment. It is crucial that intensive care is maintained and gradually withdrawn during transition from hospital discharge into the community and that self-discharge patients receive particularly comprehensive, planned follow up and aftercare (10, 17). Further, patients should be routinely contacted and followed up by phone, letter, email or in person to confirm provision and attendance of aftercare (15, 21). Follow up contact should include a component of psychoeducation to inform patients about their suicide risk and foster their engagement in treatment (22, 23). Overall, fostering trust and continuity in the relationship between the patient and provider should be a prioritized goal; discontinuity or changes in key personnel should be avoided as much as possible and some type of clinical coverage must be ensured to patients in situations when the primary provider is unavailable (5, 6, 10, 17, 21).

In contrast, literature on outpatient CoC is more limited. Extant research defines outpatient CoC as the process by which the patient and provider form and maintain a sustained partnership towards treating the patient’s diagnosis [e.g., as measured by the UPC (8, 50)]. Additional studies examined outpatient CoC among depressed patients, assessing personal continuity, team continuity and cross-boundary continuity (25). Furthermore, the well-established Norwegian model advocates for the designation of a specific team or coordinator within a community care system that would be responsible for receiving of referrals and maintaining contact with other community-based services (e.g., social services, schools, and specialty mental health services) (20).

#### Role of primary care providers and case managers in the care of suicidal patients

Multiple studies document patients who committed suicide had contact with PCPs in proximity to their death; studies document around 45% of suicide victims had contact with PCPs within a month of suicide and 77% of patients visited a general practitioner within 3 months of committing suicide (27, 29, 31, 32). Considering these alarming statistics, contemporary literature focuses on the role of PCPs in suicide assessment and prevention (32). Carrigan and Lynch (28) outlined specific guidelines for PCP’s management and continuity of care of suicide attempters such as thorough evaluation of warning signs, psychosocial circumstances and present suicide risk, providing referrals to relevant mental health resources, coordination of psychiatric aftercare for recent suicide attempters, providing encouragement and psychoeducation to combat patients’ fear or disinclination to follow through treatment referrals, follow up, medication management and monitoring of suicidal patients (28).

However, PCPs face major time constraints which impede their ability to adequately assist in the management and treatment of suicidal patients (32). Thus, efforts have led to a creation of a nonphysician, CM role (e.g., nurses, psychologists, social workers, and health care assistants) who performs these crucial tasks of CoC under the supervision of a psychiatrist (30, 32–34). Through regular in-person or phone contacts, the CM monitors the patient’s suicidal ideation [e.g., by assessing its severity using questionnaires such as the Patient Health Questionnaire [PHQ-9] or the Depression Monitoring List (51, 52)] and adherence to recommended treatment, provides brief psychotherapeutic interventions, coordinates medication management and serves as a link between the patient and PCP by providing decision support and structured reports of clinical information for the physician to subsequently provide relevant care (26, 30, 32, 34). Further, CMs assist in facilitating multidisciplinary collaborative care (i.e., coordination of medication management, referrals to specialized services) and developing treatment guidelines at primary care centers (26, 33). Contemporary evidence for the efficacy of CMs within primary care settings are elaborated in Table 1.

#### Patient follow up

Across the literature, patient follow up is defined as a major component of CoC. It refers to some type of contact with the patient following care provision (i.e., post-ED visit, hospitalization) which may be in person or in the form of a phone call, letter, text, etc. (14, 40). Present evidence notes contact should be made within about 7 days of the patient’s visit to the ED or post-discharge from hospitalization, and additional calls should be conducted on a weekly basis (41, 45, 46). Patient follow-up and monitoring should be increased during high-risk times [e.g., following hospitalization discharge, ED visit, or during transitions in care (14)]. If a patient missed a scheduled appointment, follow up is crucial to promote treatment engagement [i.e., by immediate rescheduling of a new appointment via phone, text message, email, or home visits based on clinic policy (40, 42)]. Common guidelines maintain follow up of suicidal patients should include assessment of the patient’s mood and safety, review and revision of the patient’s safety plan and problem-solving the patient’s barriers to engage in treatment (14, 44–46). Follow up must also include procedures for the provider to examine the outcomes of the contact with the patient; for example, if a CM is in touch with the patient, the CM must inform the treating physician (in person or via electronic messaging systems within the electronic medical record) if the patient is struggling with current treatment plan to ensure appropriate steps are either taken directly or discussed during team meetings (43, 44).

A prominent body of research has documented follow up of at-risk patients is efficacious in reducing suicidal behavior and death (36, 37, 41, 42, 45) as well as promoting compliance with psychotherapy (35, 41). Research on the role and efficacy of follow up on medication adherence among suicidal patients, specifically, is scarce, yet some studies among depressed patients indicate follow up contact is related to continued medication adherence (38, 39, 43). See Table 1 for a full discussion of extant evidence.

#### National and institutional guidelines and standards for CoC

Contemporary standards and best-practice guidelines of CoC have been offered both by health care organizations and major institutions [e.g., The Joint Commission, The Centers for Medicare and Medicaid Services, the VA, the APA, etc. (12)]. The VA suicide prevention standards require that patients referred to or contacting mental health services will undergo a preliminary screening within 24 h and a full-length evaluation within 2 weeks, and that patients hospitalized for suicidality be evaluated weekly during the first month post-discharge (12, 48). Further, the VA demands that patient care plans include ongoing suicidality screening and procedures for management of periods of increased risk and for follow-up of missed appointments, as well as some form of means reduction plan (12, 48). Similarly, the APA recommends promoting CoC by developing procedures to ensure availability to specific appointments for continued outpatient mental health care within a week of hospital discharge, as well as outreach and phone contact if patients do not adhere to aftercare plans (12, 15, 47). Promotion of treatment adherence is recommended by establishing a patient-provider relationship and a patient-specific treatment plan that is regularly reassessed in collaboration with the patient (47). Lastly, the American Association of Suicidology suggests providers reevaluate patients’ suicide risk prior to approving any type of referral or discharge and advocates for involvement of family and significant others in discharge and planning (i.e., routine family sessions, family should be provided explicit instructions on how to access provider and emergency contacts) (12).

## Discussion

In the present study, we conducted a narrative literature review focusing on research concerning the role of CoC in suicide prevention and treatment. Encompassing the last 26 years, our review has outlined the variability of current definitions CoC and highlighted the absence of a comprehensive conceptualization of the concept. The variability in subcategories of CoC further impact its inconsistent operationalization in research, thwarting crucial efforts of providing guidelines and implication for clinical practice. Nonetheless, the present study identifies the following components that are essential for CoC of suicidal patients: the role of CoC across multiple levels of care (i.e., following suicide-related visits to EDs, post-psychiatric hospitalization, and within outpatient care) (5, 12, 15, 20), the role of PCPs and CMs in CoC of suicidal patients (28, 30, 32), the importance of follow up contact with suicidal patients on suicidality and treatment engagement (14, 40, 45), and national\institutional guidelines for CoC of suicidal patients (47, 48).

Before we provide directions for future action and research, it is important to identify the limitations of the reviewed literature. First, the majority of research on CoC is based on psychiatric hospitalization and post-discharge management of suicidal patients (6, 7, 9). Literature on CoC of ambulatory suicidal patients is more limited. Further, the present literature fails to delineate specific guidelines for suicidal patients who are first-time users of mental health services versus long-term utilizers who are known to and familiar with the mental health care system. The majority of the literature on follow up contact with patients to promote psychotherapeutic treatment engagement and medication management is based on studies among clinically depressed (not necessarily suicidal) patients (30, 38, 39). Further, while a few RCTs exist, most studies on CoC models and their components are predominantly quasi-experimental in design, thereby seriously limiting causal inference as to the effect of CoC on preventing suicide. Lastly, while numerous guidelines and reports have been outlined by well-established organizations (e.g., VA and APA), methodological investigations of the implementation and efficacy of these protocols are scarce. Evidently, a plethora of literature exists on the subject, yet the extent of how much and how well CoC strategies are implemented in practice continue to be undetermined.

### Where do we go from here?

Based on present findings, there are numerous steps that must be taken to improve facilitation and implementation of CoC in research and clinical practice. First, the search for a unified definition must be discontinued. Just as with other complex psychological concepts (e.g., suicidality) which have been found to encompass multiple, diverse elements (e.g., suicidal ideation, attempts, and non-suicidal self-injury), CoC cannot be referred to as a single concept, as no definition is comprehensive enough to encompass the entirety of its complexity. Therefore, when referring to CoC, specific sub-type definitions should be used to guide nuanced research and clinical practice. Specifically, instead of aiming to promote a broad sense of CoC, enhancement of CoC would focus on efforts to improve different types of CoC (e.g., distinct guidelines and regulations to improve informational continuity, explicit practices to ensure provider continuity within a care system, etc.) (5, 6, 49). In addition, further research using rigorous methodology (e.g., RCTs) is needed to evaluate efficacy of extant models of CoC for suicide prevention. A comprehensive theoretical formulation of the role of CoC in suicidality is needed to provide understanding of the phenomena and to provide theory-informed guidelines for clinical practice. Explicit guidelines should be outlined both on interorganizational and intraorganizational levels. These may take the form of establishment of definitive practices for transmission of relevant care-related information of suicidal patients (e.g., summary of previous suicide risk assessment, key triggers, and safety plans), follow up through referrals and transitions of care (e.g., defining time frames and designation of key personnel responsible for patient follow up and confirmation of transition between forms of care and treatment adherence). Lastly, presently no international guidelines for CoC in suicide prevention exist; there is an evident need for internationally agreed-upon common practices. Uniform international practices and leadership would foster consultation between providers across the globe, as is commonly conducted in the medical field (53). Further, they would assist in standardizing suicide prevention practices across various cultural, social and health contexts, which may be impacted by present barriers to care (e.g., cultural beliefs and norms, mental health literary, and time and financial constraints) (54). Standardization of CoC for suicide prevention practices would assist in addressing extant service gaps, e.g., by enhancing providers’ cultural understanding and awareness raising, promoting interventions across various levels of care (e.g., community levels and care) and advancing interdisciplinary collaboration across healthcare settings (55).

## Conclusion

In conclusion, while there is abundant evidence for the centrality of CoC in suicide prevention efforts, extant literature fails to provide a comprehensive definition for this phenomenon, its measurement and operationalization which dreadfully hinders its application in clinical practice. Based on our review of research evidence on CoC for suicidality, we identified the following key components essential for CoC of suicidal patients: (1) CoC across different levels of care (e.g., after ED visit due to suicidality, post-hospitalization, across outpatient settings) (5, 12, 15, 20), (2) the role of PCPs and CMs in CoC of suicidal patients (e.g., in identification, monitoring, management and referral) (28, 30, 32), (3) the crucial role of follow up contact with suicidal patients post-treatment to assess and monitor suicidality and enhance treatment adherence (14, 45), and (4) extant national and institutional guidelines for CoC of suicidal patients (47, 48). Implications for conceptualization, facilitation and implementation of CoC for patients at risk for suicide are discussed, including a need for theoretical formulations and international practices of care that would enhance CoC across various cultural, social and healthcare settings.

## Author contributions

SA: Conceptualization, Writing – original draft, Writing – review & editing. GS: Conceptualization, Supervision, Writing – review & editing. AK: Conceptualization, Supervision, Writing – review & editing.
